# Preventive efficacy of NexGard Spectra^®^ against *Dipylidium caninum* infection in dogs using a natural flea (*Ctenocephalides felis*) infestation model

**DOI:** 10.1051/parasite/2017017

**Published:** 2017-05-12

**Authors:** Frédéric Beugnet, Leon Meyer, Josephus Fourie, Diane Larsen

**Affiliations:** 1 Merial 29 avenue Tony Garnier 69007 Lyon France; 2 ClinVet International (Pty) Ltd 9321 Universitas South Africa

**Keywords:** Dogs, *Ctenocephalides felis*, *Dipylidium caninum*, Afoxolaner, Milbemycin oxime, Prevention

## Abstract

The efficacy of a monthly oral endectocide product, NexGard Spectra^®^ (Merial), a combination of afoxolaner and milbemycin oxime, was evaluated in a flea (*Ctenocephalides felis*) challenge model for the prevention of *Dipylidium caninum* tapeworm infection in dogs. The efficacy of treatment with NexGard Spectra^®^ was assessed in 10 dogs following weekly flea infestation with metacestode naturally infected fleas and compared with that in 10 untreated control dogs. The 100 fleas deposited weekly on each dog were not removed until Day 35, allowing enough time for their ingestion. The microscopical analysis of 30 fleas from the flea batches before each weekly challenge demonstrated that 10–33% of the fleas were infected by *D. caninum* cysticercoid larvae. The arithmetic mean flea count recorded was 47.7 for the 10 untreated dogs and 0 for the 10 treated dogs at Day 35. Based on the daily collection of expelled *D. caninum* proglottids by dogs during the 70 days of the study, 70% (7/10) of the control dogs and 0% (0/10) of the treated dogs were infected with *D. caninum* (*p* < 0.0031). Through its efficacy against fleas, NexGard Spectra^®^ treatment provided indirect prevention of *D. caninum* infestation. No treatment-related adverse events were observed in dogs during this study.

## Abstract (fr)

L’efficacité d’un médicament endectocide oral mensuel, NexGard Spectra^®^ (Merial), une combinaison d’afoxolaner et de milbémycine oxime, a été évaluée dans un modèle d’infestation par des puces (*Ctenocephalides felis*) pour la prévention de l’infection du ténia *Dipylidium caninum* chez les chiens. L’efficacité du traitement de 10 chiens avec NexGard Spectra^®^ a été évaluée suite à une infestation hebdomadaire avec des puces infectées naturellement par des métacestodes et comparée à 10 chiens témoins non traités. Les 100 puces déposées chaque semaine sur chaque chien n’ont pas été enlevées jusqu’au jour 35, ce qui donnait suffisamment de temps pour leur ingestion. L’analyse microscopique de 30 puces des lots de puces avant chaque épreuve hebdomadaire a démontré que 10 à 33 % des puces étaient infectées par les larves cysticercoïdes de *D. caninum*. La moyenne arithmétique des comptages de puces était de 47,7 et de 0 au jour 35, pour les 10 chiens non traités et les 10 chiens traités, respectivement. Sur la base de la collecte quotidienne des proglottis de *D. caninum* expulsés par les chiens au cours des 70 jours de l’étude, 70 % (7/10) des chiens témoins et 0 % (0/10) des chiens traités ont été infectés par *D. caninum* (*p* < 0,0031). Grâce à son efficacité contre les puces, le traitement par NexGard Spectra^®^ a fourni une prévention indirecte contre l'infestation à *D. caninum*. Aucun effet indésirable lié au traitement n’a été observé chez les chiens au cours de cette étude.

## Introduction

The cat flea, *Ctenocephalides felis felis* Bouché, 1835, is widespread all over the world [10, 14, 15, 23]. The infestation of cats and dogs, but also many other mammals including humans, is very common. Flea infestations often induce clinical signs in dogs such as pruritus, hair loss and flea allergy dermatitis [14, 19], and increase the risk of pathogen transmission including *Bartonella* spp*.* and *Rickettsia felis* [1]. The cat flea is also the intermediate host of a tapeworm, *Dipylidium caninum* (Linnaeus, 1758) [7, 8]. The tapeworm life cycle starts with the flea larvae ingesting the proglottids or egg packets containing the eggs of *D. caninum* in the environment; the eggs then hatch and the hexacanth embryos infect the flea larva. The hexacanth embryos develop in immature flea stages in 9 to 19 days to non-infective metacestodes that stay quiescent in the flea pupae [6, 20, 21]. Following the original description by Venard [24], Pugh identified seven morphological stages of the *Dipylidium* metacestode during its maturation and preferred to avoid using the term cysticercoid larvae [20, 21]. Once the adult flea emerges and infests a host, the metacestode quickly reactivates and matures into an infective metacestode stage in the flea within 1–3 days [20, 21, 24]. This development is temperature dependent (with an optimal temperature of 32 °C) and is related to the presence of the fleas on the host skin. Carnivores become infected through the ingestion of fleas containing infective cysticercoid larvae, and adult *D. caninum* are typically formed within 2–3 weeks and can stay alive in their hosts for up to 3 years. At that time, proglottids are eliminated by infected carnivores.

*Dipylidium caninum* is common worldwide, infecting both cats and dogs, and is zoonotic, even though human infection is very rare [7]. In a recent publication based on the collection of fleas on 435 cats and 178 dogs in Europe, 5.2% of 732 *C. felis* and 2.2% of 1969 *C. felis* were found to be infected by *D. caninum*, which indicates that this tapeworm is probably more common than veterinarians may think [5]. The main difficulty in diagnosis is the poor sensitivity of coproscopical examination of faeces because proglottids can leave their host actively not during defecation, and because they are mainly located on the surface of faeces that they can also leave [5–7].

The regular use of an effective anti-flea insecticide is important to protect pets against the various pathogenic effects related to flea infestation [10, 15]. Many insecticides and/or acaricides are available for use in cats and/or dogs in a variety of formulations [4]. It has previously been demonstrated with a fipronil-(S)-methoprene spot-on formulation (Frontline Combo^®^, Merial) that applying topical insecticide that kills fleas fast enough would prevent the transmission of *D. caninum* [2]. The preventive efficacy is based on the time needed for the metacestode to mature and become infective to dogs or cats after flea ingestion, which was estimated between 24 and 36 h mainly based on temperature increase on the host’s skin [20, 21]. This maturation of the metacestodes can be discerned by morphological changes, as originally described by Venard in 1938 [24]. In the present study, we assessed the protection against *D. caninum* infection conferred by the oral administration of NexGard Spectra^®^ (Merial) to dogs. NexGard Spectra^®^ is an oral chewable formulation containing afoxolaner, an insecticidal-acaricidal compound of the isoxazoline family, and milbemycin oxime, a nematodicidal compound [17]. It is intended to protect dogs against flea and tick infestation, and to deworm against gastro-intestinal nematodes as well as some other nematode infections (*Dirofilaria immitis*, *Angiostrongylus vasorum*) [9, 12, 16, 22]. Since milbemycin oxime is only nematodicidal, no efficacy is claimed against tapeworms. However, afoxolaner is used at 2.5 mg/kg minimum dose to provide rapid curative and sustained efficacy against fleas [3]. Based on several publications, the sustained speed of kill of afoxolaner against fleas ranges from 12 to 24 h for the duration of the month [3, 9]. Our hypothesis was that *D. caninum-*infected fleas should be killed before the maturation of the metacestodes, therefore the treatment would indirectly prevent dogs against this tapeworm infection.

## Materials and methods

### Production of fleas infected by *Dipylidium* metacestodes

To assess the prophylactic effect of anti-flea treatment, it was first necessary to produce *Dipylidium*-infected fleas. The methodology was described previously [2, 13]. In order to do this, donor dogs infected with *D. caninum*, as confirmed by proglottid observation in faeces, were also infested with *C. felis* fleas. Flea eggs and shed proglottids were collected in a paper-covered pan below the cages every 24 or 48 h. The contents were sieved to remove gross debris. The sieved material containing flea eggs, *Dipylidium* proglottids and egg packets, was placed in an incubator (at 25 °C) in Petri dishes. The flea eggs started hatching after approximately 2–3 days, and the flea larvae were maintained with the sieved material for another 2 days (i.e., up to about 6 days after sieving). The mixture was then transferred to a classic flea breeding unit, containing a mixture of sand and crushed dried cat food, to ensure that the flea larvae could feed and develop adequately to pupae and newly emerged fleas within 2 weeks. *Dipylidium* metacestode development occurred in parallel to flea development.

### Design of the studies

This study was conducted in accordance with the VICH GL9 (June 2000) guidelines on Good Clinical Practice [11].

The experimental unit was designed in compliance with the South African National Standard “SANS 10386:2008 *The care and use of animals for scientific purposes*”. The protocols were submitted to the ClinVet Animal Ethics Committee (CAEC).

This was a parallel group, blind, randomised, negative control efficacy study (Table 1) [18]. It included two groups of 10 dogs each, selected from an enrolled group of 24 dogs. The four dogs with the lowest body weights were removed. The groups consisted of 10 dogs in the negative control group and 10 dogs in the NexGard Spectra^®^ treated group.

**Table 1.** Experimental design.

Acclimatisation	Treatment	Flea infestation (*D. caninum-*infected fleas)	Flea count and removal	Faecal and cage floor examination
Days – 7	Day 0	Days 7, 14, 21 and 28	Day 35	Daily from Days 28–70

The study followed a randomised block design. The 20 selected dogs were ranked within sex in descending order of individual animal body weight and subsequently allocated to 10 blocks of two dogs each. Within blocks, dogs were randomly allocated to the two groups.

Assignment of codes (codes A and B) to the study groups and administration of the treatment was the responsibility of non-blinded personnel. All other people involved in the study were blinded to the group allocation. Permission to deblind the study after study termination was given by the sponsor representative via e-mail received on 11 November 2016.

The dogs included in the study were mixed breed dogs, originating from the facility, ≥ 6 months of age. These dogs had not been treated with a long-acting topical or systemic acaricide/insecticide during the 12 weeks preceding Day 0, and the females were not pregnant. They were acclimatised to the study site for 7 days before the start, and they were clinically healthy as determined by a veterinarian on Day −7. None of the dogs were removed from the study prior to study termination.

The dog cages were environmentally controlled for temperature (20 °C ± 4 °C). The floor size of each dog cage was 2.0 m × 3.0 m. On Day 35, after the final flea count and removal, all dogs were moved to an outside unit. The new dog cages consisted of a 1.70 m × 0.7 m enclosed sleeping area and an outside run of 1.70 m × 3.0 m. A roof covered the kennels and the dogs were not exposed to rain.

The dogs were kept individually and no physical contact between them was possible. However, dogs still had visual and auditory contact with conspecifics. They were fed once a day according to the food manufacturer’s recommendation.

The NexGard Spectra^®^ treatment was administered once on Day 0 at the doses indicated in Table 2. The purpose was to administer a dose as close as possible to the minimum dose of afoxolaner (2.5 mg/kg) and milbemycin oxime (0.5 mg/kg), so that all dogs received a similar concentration of the active ingredients in mg/kg.

**Table 2.** Administered tablet doses per individual dog in treated group.

Group 2 dogs		Tablets to be administered (g)						
Animal ID	Body weight (kg)	0.5	1	2	4	8	Afoxolaner dose (mg)	Milbemycin dose (mg)
1FD 220	16.62	1	0	1	0	0	46.88	9.38
2AC DC2	19.26	0	1	1	0	0	56.25	11.25
4DA C4C	17.20	1	0	1	0	0	46.88	9.38
5A3 1B0	12.40	0	0	1	0	0	37.5	7.5
5B3 502	16.36	1	0	1	0	0	46.88	9.38
5CB 26C	12.80	0	0	1	0	0	37.5	7.5
5E0 B03	14.60	0	0	1	0	0	37.5	7.5
6D5 307	18.40	1	0	1	0	0	46.88	9.38
B2B 55A	17.80	1	0	1	0	0	46.88	9.38
E19 260	16.80	1	0	1	0	0	46.88	9.38
NexGard spectra^®^					Size of chewable tablets (g)		Afoxolaner (mg)	Milbemycin oxime (mg)
Chewable tablets for dogs 2.0 kg–3.5 kg					0.5		9.38	1.88
Chewable tablets for dogs > 3.5 kg–7.5 kg					1		18.75	3.75
Chewable tablets for dogs > 7.5 kg–15.0 kg					2		37.50	7.50
Chewable tablets for dogs > 15.0 kg–30.0 kg					4		75.00	15.00
Chewable tablets for dogs > 30.0 kg–60.0 kg					8		150.00	30.00

### Experimental infestation with *D. caninum*-infected fleas

All dogs were experimentally infested with 100 *C. felis* fleas that were infected with a dog strain of *D. caninum,* on Days 7, 14, 21 and 28. Three flea batches were used during the study. A sample of 30 fleas was examined before each weekly flea challenge. Each of the 30 fleas was dissected and then examined microscopically to determine the presence or absence of *D. caninum* metacestodes (Table 3). For the flea challenges, the fleas were deposited on the dorsal line of the dogs on each of the infestation days, and the dogs were allowed to groom freely. Fleas were not combed, counted nor removed until Day 35.

**Table 3.** Flea batches and infection rates used per infection.

Day	Parasite	Batch number	1Prevalence of metacestode infection (%)	2Mean intensity of metacestodes per infected flea
7	*Ctenocephalides felis –* infected with *Dipylidium caninum* (dog origin)	270716	10	13
14		170816	27	3.5
21		170816	17	1.6
28		310816	33	5.2

1 Prevalence = number of infected fleas/30 examined fleas per batch per week.

2 Mean intensity of metacestodes = total number of metacestodes recovered/number of infected fleas.

### Clinical assessment

A veterinarian conducted a clinical examination on all dogs during acclimatisation for enrollment on Day −7 and then again on Days −2, 35 and 70. All the dogs were observed daily by technicians from Day −7 to Day 70 for their general health.

Based on the prepatent period of *D. caninum* [6, 7], the cage floors, sleeping areas and faeces were examined daily from Days 28 to 70 to determine the possible presence of cestode proglottids. The cage floors and sleeping areas were carefully scrutinised daily by a technician to find proglottids, whereas the faeces were collected and diluted in water for complete observation under binocular magnifier. As soon as a dog was diagnosed as infected, it was removed from the study and dewormed with an anthelmintic containing praziquantel (5 mg/kg) (Milbemax^®^, Elanco).

Based on the prepatent period of approximately 21 days, and the last flea infestation on Day 28, it was considered that taking a margin of 21 more days, after Day 70, there was no biological possibility that a negative dog would become positive.

### Efficacy criteria

The assessment criterion was the presence or absence of *D. caninum* infection by daily macroscopical examination of both faeces and cage floors from Day 28 to Day 70.

Percentage efficacy at the end of the study was calculated using the following formula:

$$\mathrm{Efficacy}\;\left(\%\right)=\;100\;\times \left(T_{c}\;-\;T_{t}\right)T_{c},$$

where:

*T*_c_ = Total number of infected dogs in the negative control group;
*T*_t_ = Total number of infected dogs in the treatment group.

SAS Version 9.3 TS Level 1M2 was used for all the statistical analyses. The absence and presence of proglottids were compared between the groups using a χ^2^ test or a Fisher’s exact test when one or more of the cells had a frequency of five or less. The level of significance of the formal tests was set at 5%, all tests were two-sided.

The number of fleas collected from dogs in both groups at Day 35 was considered an informative criterion.

## Results

On Day −2, the mean body weight of dogs was 16.76 kg (ranging from 12.74 kg up to 22.00 kg) for the negative control group, and 16.22 kg (ranging from 12.40 kg up to 19.26 kg) for the NexGard Spectra^®^ treated group (*p* = 0.6462), indicating homogeneity at the time of inclusion.

One dog in the negative control group had bloody loose faeces once on Day 29. As the faeces of the dog were normal afterwards and the dog showed no other clinical signs (fever, pain, etc.), no concomitant therapy was required.

On Day 35, one dog from the treated group had erythema on the abdomen and inguinal area. The signs were mild and resolved spontaneously without therapy.

Another dog in the treatment group showed signs of hair loss on the left and right flanks during the study on Day 43. The dog received concomitant topical essential oil therapy (Dermoscent^®^, LDCA) and the skin condition started to improve. The hair loss was considered not related to the treatment.

The individual dog cage floors and faeces were examined daily for proglottids from Days 28 to 70. Seven out of the 10 dogs in the negative control group were found to be positively infected with *D. caninum*: one dog on Day 35, one on Day 37, one on Day 38, one on Day 43, one on Day 51, one on Day 63 and one on Day 65.

None of the 10 dogs in the treated group were found to be infected with *D. caninum* (Table 4).

**Table 4.** Number of dogs infected by *Dipylidium caninum*.

	Untreated control Group	NexGard Spectra^®^ treated Group		
Day	Infected dogs	Infected dogs	Percentage efficacy	
28–70	7/10 Days of diagnosis: 35, 37, 38, 43, 51, 63 and 65	0/10	100.0	*p* < 0.0031

In this study, a single treatment with orally administered NexGard Spectra^®^ was thus 100% effective in preventing infection with *D. caninum* tapeworms in 10 dogs, after four weekly infestations with 100 fleas from a population infected by *D. caninum*.

A statistically significant difference was observed between the negative control group and the treatment group (*p* < 0.0031).

An arithmetic mean of 47.7 fleas were recovered from the untreated control dogs, whereas no fleas were recovered from the treated group on Day 35 (Table 5), corresponding to 100% efficacy against fleas at Day 35.

**Table 5.** Fleas recovered from the control and treated dogs on Day 35.

Untreated control group Animal ID	Number of removed fleas on Day 35	Treated group Animal ID	Number of removed fleas on Day 35
698 456	38	6D5 307	0
DF7 C6D	23	1FD 220	0
4DD 2E5	36	5A3 1B0	0
4EF 726	41	E19 260	0
5D1 0EA	78	5CB 26C	0
E17 4A6	70	2AC DC2	0
6D5 271	76	B2B 55A	0
5B3 DA9	43	5B3 502	0
287 685	18	4DA C4C	0
86A C4C	54	5E0 B03	0
Average flea count	47.7	Average flea count and efficacy	0 (100%)

Supplementary files illustrating the study are available at http://www.parasite.org/10.1051/parasite/2017017/olm

## Discussion

As previously published [2, 13], the experimental flea-infection model worked well, producing a population of *Dipylidium* metacestode-infected fleas, at a rate of 10–33%. The production of infected fleas enables the design of specific studies assessing treatment and prevention against fleas and tapeworms in cats or dogs. Under natural field conditions, the infection rate of fleas seems to be quite low, from 2% to 5% of *Dipylidium-*infected fleas, based on recent flea surveys in Europe [5]. It can therefore be considered that the flea challenges used in this study were higher than the natural risk [2, 13]. As already demonstrated for two topical products (Frontline Combo^®^ and Certifect^®^, Merial) and one collar (Seresto^®^, Bayer), the oral administration of NexGard Spectra^®^ to dogs was 100% effective in preventing infections with *D. caninum*, despite the fact that 100 fleas were applied to each dog four times, at weekly interval (Days, 7, 14, 21 and 28). In order to demonstrate that this efficacy would be obtained whatever the weight of the treated dogs, the minimum effective dose was used (i.e. as close as possible to 2.5 mg/kg afoxolaner and 0.5 mg/kg milbemycin) instead of the commercial weight range. Animals infested with fleas groom themselves and ingest fleas. Dogs appear to ingest fewer fleas than cats, which could explain why all cats were found infected by *Dipylidium* in a previous study [2], whereas only seven out of eight dogs were infected during a 2-month period of flea infesting challenges using 250 fleas per challenge. In the present study, 7 out of 10 control dogs were infected after the 4 flea challenges using 100 fleas each. In order to provide protection against *D. caninum* infection, the anti-flea treatment needs to kill fleas before the maturation of the metacestodes [2, 20, 21]. Based on available data, it seems that metacestodes need 24–36 h to become infective for the definitive host, which is temperature-related [20, 21]. We assumed that by killing fleas within 12–24 h during the whole month [3, 9], even if killed fleas were ingested, afoxolaner provided an indirect protective effect against *D. caninum* infection.

In this study, the efficacy criterion was based on the collection of *Dipylidium* proglottids, either on dog’s faeces or on cage floors. Based on previous studies, this was demonstrated to be sensitive enough to recover all infected animals [2, 3]. Microscopical coproscopy is known to have poor sensitivity and would bring no any additional benefit to the experimental design used here. *Dipylidium* infection can also be assessed by polymerase chain reaction (PCR); it is of interest for epidemiological surveys based on flea analyses [5]. The sensitivity of the PCR technique on faeces may be impaired by inhibiting factors and high bacterial contamination. As the preventive efficacy is based on individual analysis, pooled PCR analysis of faeces would be of no interest here.

In this controlled study, protection was complete for the whole month. In addition to providing monthly flea and tick control and a deworming effect against roundworms, hookworms, whipworms, heartworm larvae and lungworm [12, 16, 17, 22], the regular monthly use of NexGard Spectra^®^ should indirectly help to prevent the infection of dogs by *D. caninum*.

## Conflict of interest

 The first and last authors are employees of Merial, which produces the veterinary drugs used in these studies.

NexGard Spectra^®^ and Frontline Combo^®^ are registered trademarks of Merial. All other marks are the property of their respective owners. This document is provided for scientific purposes only. Any reference to a brand or trademark herein is for information purposes only and is not intended for any commercial purposes or to dilute the rights of the respective owners of the brand(s) or trademark(s).

## Supplementary Material

Supplementary Material
