# Supplementary material for: Preventive efficacy of NexGard Spectra® against Dipylidium caninum infection in dogs using a natural flea (Ctenocephalides felis) infestation model
Source: Parasite. 2017 May 12;24:16. doi: 10.1051/parasite/2017017 (PMC5432962; doi:10.1051/parasite/2017017)
Supplement: Supplementary Material [file parasite-24-16-s1.pdf]

Supplementary Material

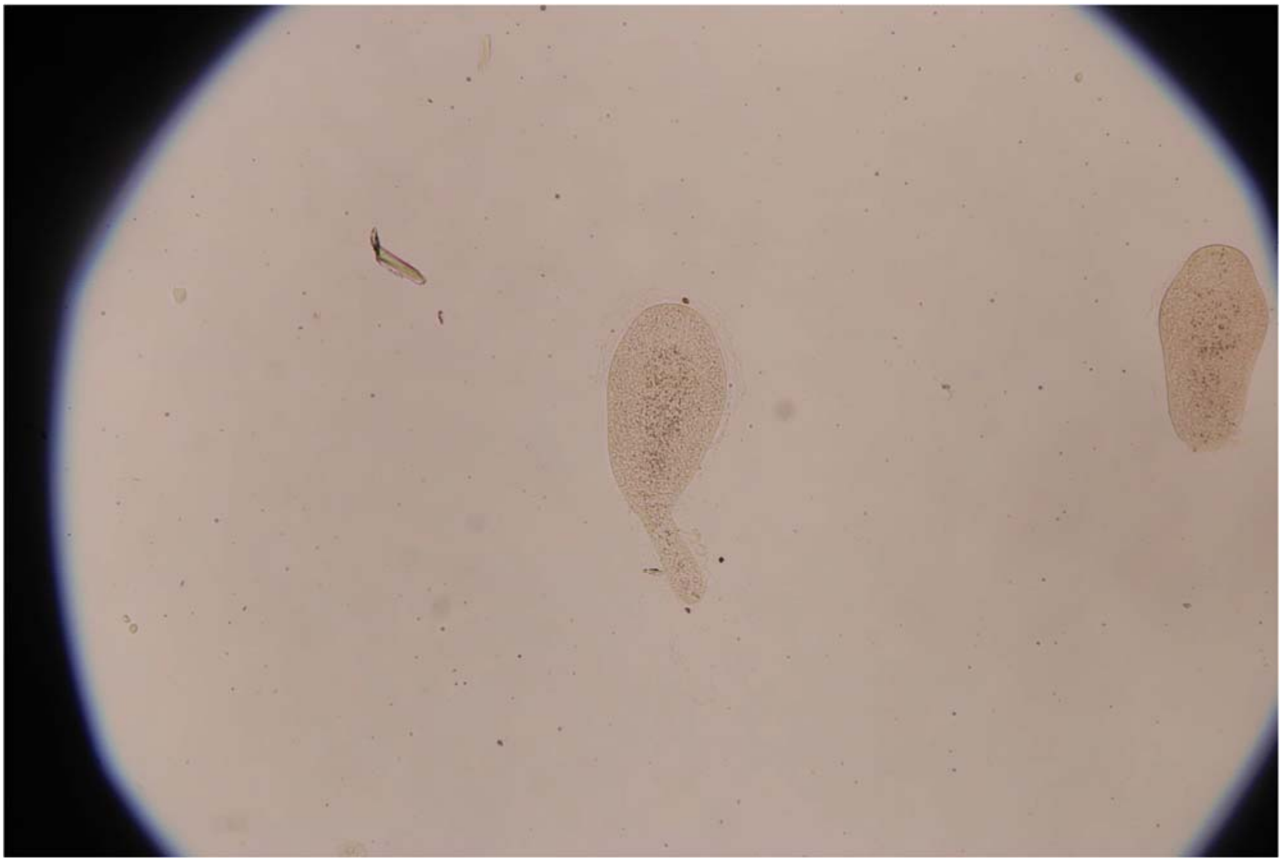

Fig. S1. Immature Dipylidium caninum metacestode observed after dissection of a newly emerged flea in water. The hexacanth hooks from the embryo migrate into a tail which is released. The metacestode thereafter appears ovoid. No suckers, no rostellum, and no hooklets are yet formed.

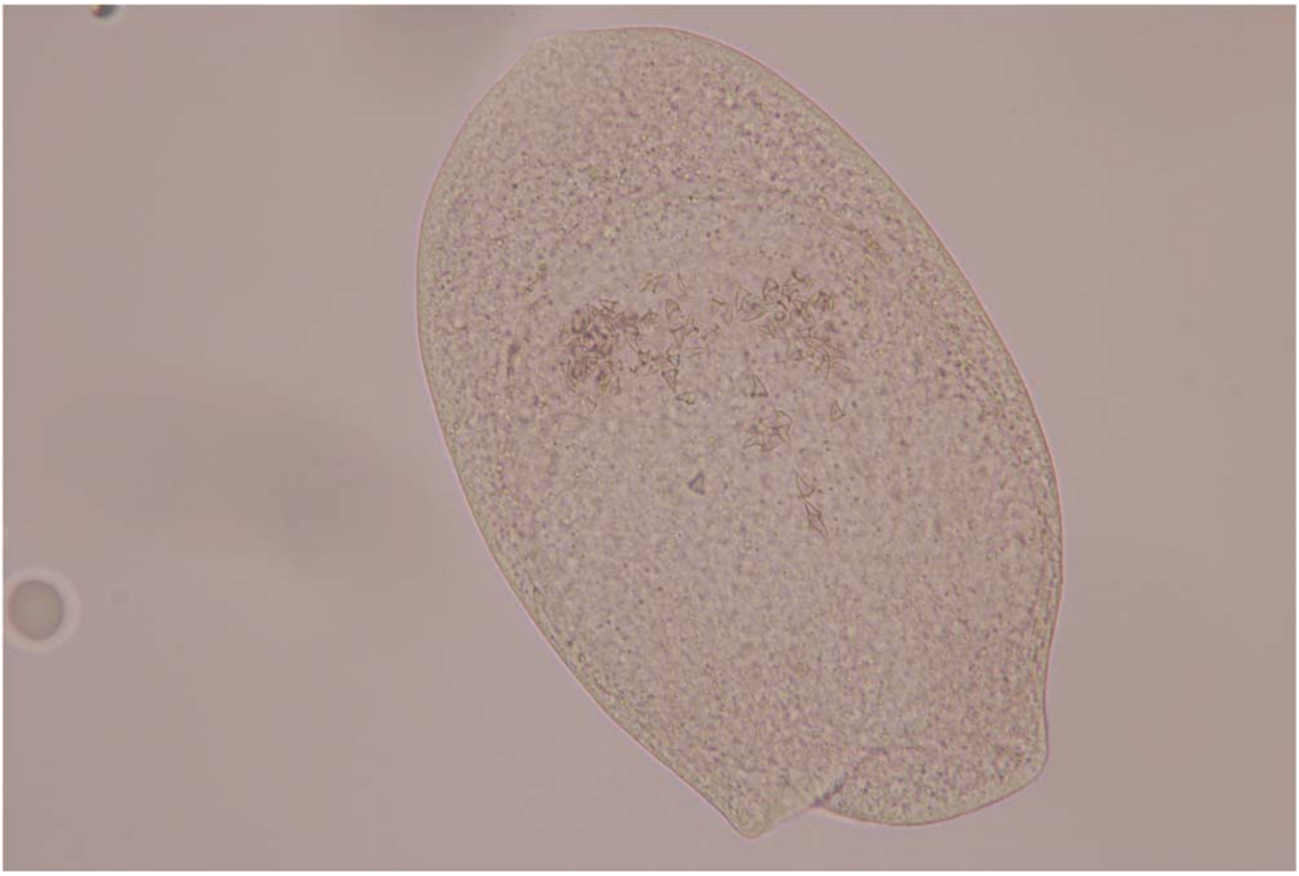

Fig. S2. Immature Dipylidium caninum metacestode in development, observed after dissection of a newly emerged flea. The suckers are in formation at one apex of the metacestode. The future hooklets appear in the middle of the larva.

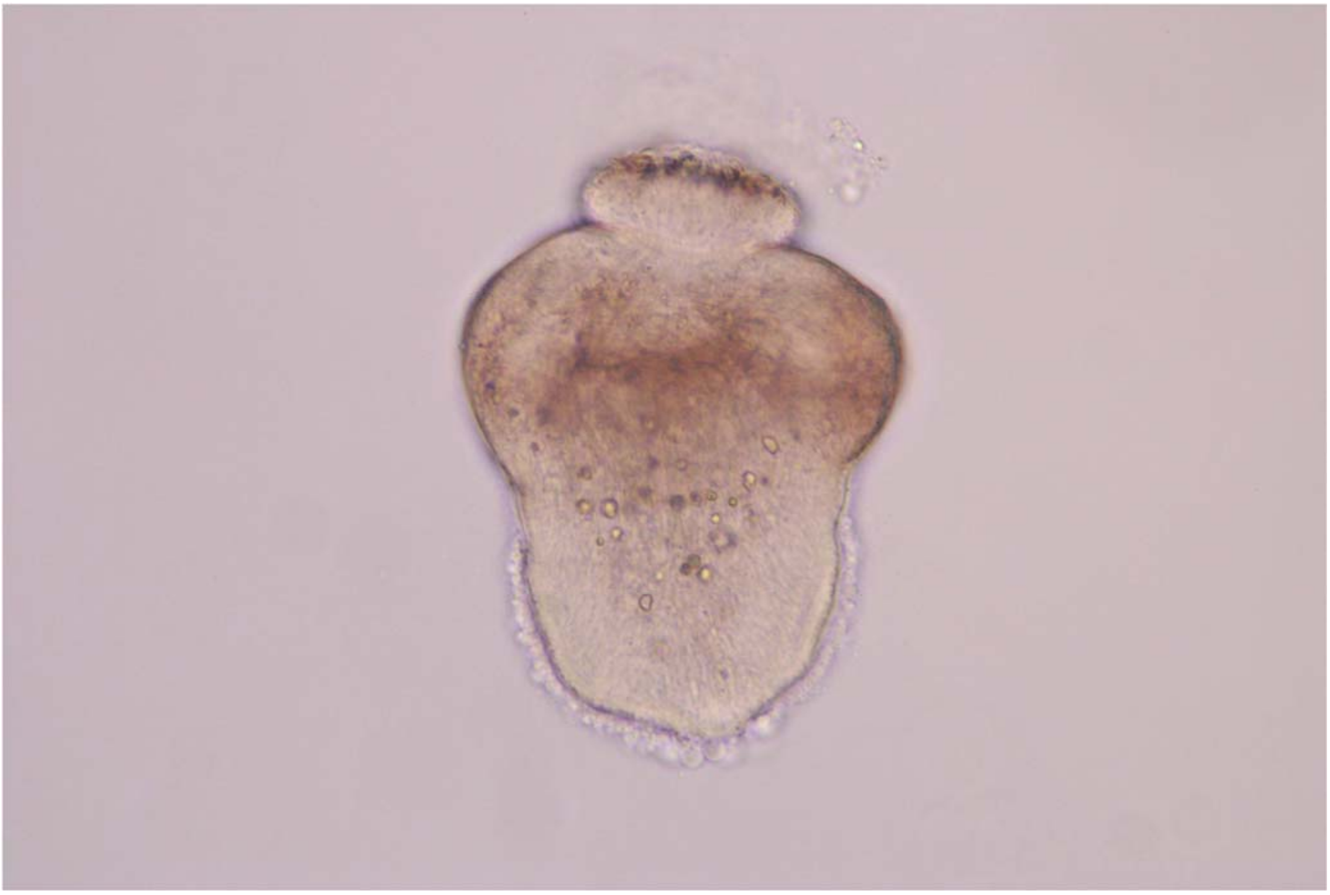

Fig. S3. Mature infective Dipylidium caninum metacestode, observed after dissection of a flea collected on the host after 36 hours. The rostellum with hooklets is everted. The suckers are clearly visible. This larval stage is infective for dogs and cats after ingestion.

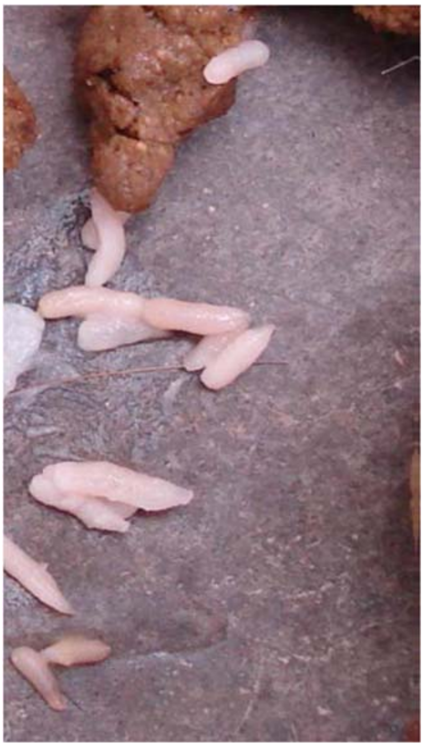

Fig. S4. Dipylidium caninum proglottids visible on dog faeces.

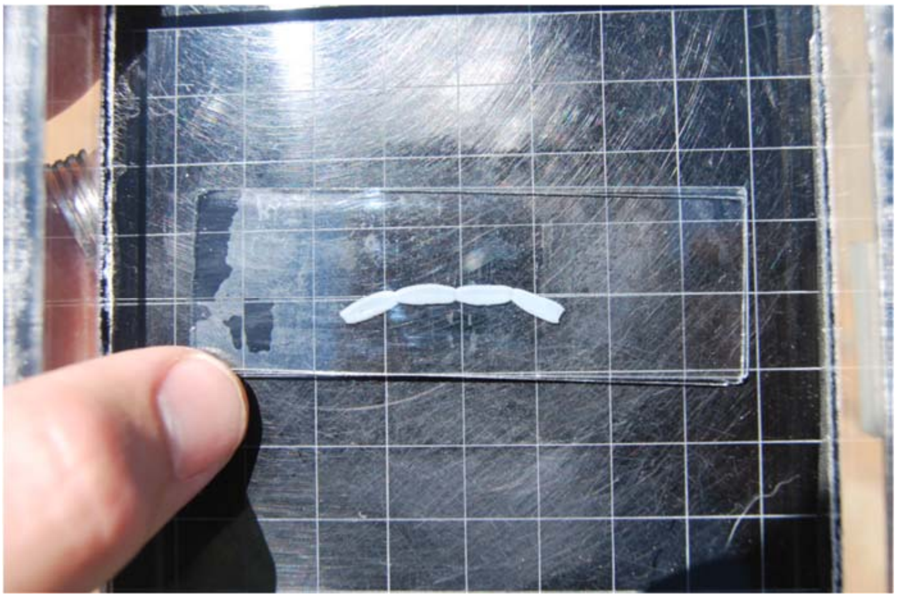

Fig. S5. Dipylidium caninum segments containing 4 proglottids recovered from the cage floor.
